# Metabolism and growth inhibition of four retinoids in head and neck squamous normal and malignant cells

**DOI:** 10.1054/bjoc.2001.1952

**Published:** 2001-08

**Authors:** I Klaassen, R H Brakenhoff, S J Smeets, G B Snow, B J M Braakhuis

**Affiliations:** Section Tumor Biology, Department of Otolaryngology/Head and Neck Surgery, University Hospital, *Vrije Universiteit*, PO Box 7057, Amsterdam, MB, 1007, The Netherlands

**Keywords:** head and neck cancer, isotretinoin, keratinocytes, metabolism, retinoid, squamous

## Abstract

Isotretinoin (13- *cis* -retinoic acid, 13cRA) has proven to be active in chemoprevention of head and neck squamous cell carcinoma (HNSCC). Moreover, both all-*trans*-retinoic acid (ATRA) and 13cRA induce objective responses in oral premalignant lesions. After binding of retinoids to retinoic acid receptors (RARs and RXRs) dimers are formed that are able to regulate the expression of genes involved in growth and differentiation. We compared the metabolism and level of growth inhibition of 13cRA with that of ATRA, 9cRA and retinol in four HNSCC cell lines and normal oral keratinocyte cultures (OKC). These retinoid compounds are known to bind with different affinities to the retinoic acid receptors. We observed that all retinoids were similar with respect to their capacity to induce growth inhibition. One HNSCC line could be ranked as sensitive, one as moderately sensitive and the remaining two were totally insensitive; OKC were moderately sensitive. The rate at which the cells were able to catabolize the retinoid was similar for all compounds. Retinoid metabolism in HNSCC cells resulted in a profile of metabolites that was unique for each retinoid. These metabolic profiles were different in OKC. Our findings indicate that differences in retinoid receptor selectivity of these retinoids do not influence the level of growth inhibition and rate of metabolism. © 2001 Cancer Research Campaign http://www.bjcancer.com


*British Journal of Cancer* (2001) **85**(4), 630–635
© 2001 Cancer Research Campaign
doi: 10.1054/ bjoc.2001.1952, available online at http://www.idealibrary.com on IDEAL

http://www.bjcancer.com

### Metabolism and growth inhibition of four retinoids in head and neck squamous normal and malignant cells

**I Klaassen, RH Brakenhoff, SJ Smeets, GB Snow and BJM Braakhuis**

Section Tumor Biology, Department of Otolaryngology/Head and Neck Surgery, University Hospital *Vrije Universiteit*, PO Box 7057, 1007 MB Amsterdam, The Netherlands

**Summary** Isotretinoin (13-*cis*-retinoic acid, 13cRA) has proven to be active in chemoprevention of head and neck squamous cell carcinoma (HNSCC). Moreover, both all-*trans*-retinoic acid (ATRA) and 13cRA induce objective responses in oral premalignant lesions. After binding of retinoids to retinoic acid receptors (RARs and RXRs) dimers are formed that are able to regulate the expression of genes involved in growth and differentiation. We compared the metabolism and level of growth inhibition of 13cRA with that of ATRA, 9cRA and retinol in four HNSCC cell lines and normal oral keratinocyte cultures (OKC). These retinoid compounds are known to bind with different affinities to the retinoic acid receptors. We observed that all retinoids were similar with respect to their capacity to induce growth inhibition. One HNSCC line could be ranked as sensitive, one as moderately sensitive and the remaining two were totally insensitive; OKC were moderately sensitive. The rate at which the cells were able to catabolize the retinoid was similar for all compounds. Retinoid metabolism in HNSCC cells resulted in a profile of metabolites that was unique for each retinoid. These metabolic profiles were different in OKC. Our findings indicate that differences in retinoid receptor selectivity of these retinoids do not influence the level of growth inhibition and rate of metabolism. © 2001 Cancer Research Campaign http://www.bjcancer.com

**Keywords**: head and neck cancer; isotretinoin; keratinocytes; metabolism; retinoid; squamous

Retinoic acid is an active metabolite of retinol (ROL, vitamin A) that plays an important role in growth and differentiation of a variety of cell types (Gudas et al, 1994). Retinoids also inhibit the growth of cancer cells (Lotan, 1980) and are useful in the treatment and prevention of human cancer (Bollag and Holdener, 1992). In head and neck squamous cell carcinoma (HNSCC) 13-*cis*-retinoic acid (13cRA) was successful in the prevention of second primary tumours (Hong et al, 1990). Retinoids demonstrate the ability to induce objective responses in oral leukoplakia (Hong and Itri, 1994). This is a whitish and reddish plaque in the epithelial lining in the oral cavity that have a relatively high risk to become malignant. For 13cRA a response rate (based on both partial and complete responses) varying between 55% and 87% has been reported (Lippman et al, 1988; Hong and Itri, 1994). All-*trans*-retinoic acid (ATRA) was shown to be active in 59% of leukoplakia patients (Koch, 1978). The activity of retinoids, however, is associated with serious side effects and intrinsic and acquired resistance does occur (Hong and Itri, 1994). Acquired resistance to ATRA in leukemia was found to be a consequence of enhanced catabolism of ATRA (Muindi et al, 1994). In contrast, an inverse relationship was found between intrinsic ATRA resistance and ATRA catabolism in HNSCC (Braakhuis et al, 1997; Klaassen et al, 2001a). This paradoxical finding suggests that the molecular mechanisms underlying RA-resistance in HNSCC are different. The possible mechanisms explaining (the lack of) the effect of retinoids have not been elucidated but are thought to be mediated through retinoid binding to specific nuclear retinoid receptors, with modulation of the expression of genes involved in growth and differentiation (Oridate et al, 1996). Recent data show that in HNSCC especially the expression of RAR-γ is important for retinoid induced growth inhibition (Le et al, 2000; Klaassen et al, 2001b).

ATRA is a naturally occurring compound that shows a much more avid binding to retinoic acid receptors than 13cRA (Åström et al, 1990). Whereas ATRA preferentially binds retinoic acid receptors (RARs), its stereoisomer 9-*cis*-RA (9cRA) has a high binding affinity for RARs as well as retinoid X receptors (RXRs) (Heyman et al, 1992). Details on their binding and transactivating activity has been reported earlier in full detail (Allenby et al, 1993; Sun et al, 1997). Related to differences in receptor binding affinities between the three stereoisomers, different effects on growth and metabolism may be expected.

Differences in metabolic rates and/or type of metabolites formed between the various retinoid compounds may determine the growth inhibiting capacities of these compounds. One important route of ATRA metabolism consists of hydroxylation at position 4 of the cyclohexenyl ring to form 4-hydroxy-RA, which is readily oxidized to 4-oxo-RA. Other metabolic pathways of ATRA include isomerization, decarboxylation and glucuronidation processes (Rockley et al, 1980; Roberts et al, 1980; Sass et al, 1994). In vitro, 4-hydroxylation of ATRA is mediated by a cytochrome P450 (CYP)-dependent mono-oxogenase system that requires NAPDH and oxygen (Roberts et al, 1980).

The present study was undertaken to investigate to what extent a panel of retinoids, including 13cRA, is able to inhibit growth of HNSCC cells and cultures of normal oral keratinocytes (OKC). Growth inhibition was related to retinoid turnover and the pattern of metabolites formed.

*Received 11 January 2001*
*Revised 30 May 2001*
*Accepted 31 May 2001*

*Correspondence to:* BJM Braakhuis

### MATERIALS AND METHODS

### Chemicals

13cRA and retinol (ROL) were obtained from Sigma (St. Louis, MO), ATRA from Acros Chimica (Geel, Belgium); 4-oxo-all-*trans*-RA (4-oxo-RA) and 4-oxo-13cRA were gifts from Dr U.H. Wiegand (Hoffmann-la Roche, Basel, Switzerland); 4-hydroxy-RA (4-OH-RA) and 18-hydroxy-RA (18-OH-RA) were gifts from Dr L. Foley (Roche Pharmaceuticals, Nutley, NJ). All compounds were dissolved in dimethyl sulfoxide (DMSO) and stored as $10^{-3}$ mol $l^{-1}$ stock at –80˚C under nitrogen. For each experiment, working dilutions were freshly prepared in the appropriate cell culture medium. Final DMSO concentration was always lower than 0.1% and did not affect cell growth. All handling with retinoids was performed in subdued light and in the presence of 0.1% BSA to prevent absorption to plastics (Klaassen et al, 1999).

### Cells and culture conditions

Primary OKC were obtained from the uvulas of non-cancer patients who underwent uvulopalatopharyngoplasty. The mucosal layer was stripped from the tissue and cells were isolated and cultured as described previously (Reid et al, 1997) in 6-well culture plates in keratinocyte growth medium (KGM) (Life Technologies, Paisley, UK) supplemented with growth factors (BPE and rEGF), gentamycin sulphate (final concentration 5 µg $ml^{-1}$) and amphotericin B (final concentration 0.5 µg $ml^{-1}$) (all from Life Technologies). At 70% confluency, primary cultures of keratinocytes were subcultured and plated at a density of $10^5$ cells/well. Keratinocytes were used for experiments at passage 3, when they reached 70–80% confluence. Human HNSCC cell lines UM-SCC-35, –22A and –14C were provided by Dr TE Carey (University of Michigan, Ann Arbor, MI) and are described elsewhere (Carey, 1990). An RA-resistant subline, UM-SCC-35R, was established from the RA-sensitive UM-SCC-35 cell line by exposing it to increasing concentrations of ATRA ($10^{-8}$ to $10^{-6}$ mol $l^{-1}$) during a period of 8 months. HNSCC cells were maintained in Dulbecco's Modified Essential Medium (DMEM, Life Technologies) supplemented with 5% FCS (ICN Biomedicals, Irvine, UK) and 50 units $ml^{-1}$ penicillin, 50 µg $ml^{-1}$ streptomycin (Life Technologies).

### Growth inhibition assays

The dose response effect on cell proliferation was determined with the 'SRB-assay'. Details of the assay have been described previously (Braakhuis et al, 1993). In short, cells (1000–4000 per well) were plated in 96-well plates in DMEM/5% FCS or KGM/0.1% BSA for HNSCC cell lines and oral keratinocyte cultures, respectively, and were allowed to grow for 72 h (the 'lag phase'). After this phase the medium was replaced by medium containing the appropriate retinoid with a final concentration ranging from $10^{-9}$ to $10^{-6}$ mol $l^{-1}$. Growth was assayed after another 72 h incubation (the 'log phase'), by staining the cellular protein with sulphorhodamine B (SRB; Sigma) and spectrophotometric measurement of the absorption at 540 nm with a microplate reader (Labsystems Multiskan, Helsinki, Finland). Growth of untreated cells during the log phase must at least have doubled its initial value.

### Metabolism experiments

Keratinocytes and HNSCC cells were plated at a density of $10^5$ cells per well in 6-well plates. Upon 70–80% confluence, the medium was removed and replaced by medium containing 1 µM RA, being KGM with 0.1% BSA for keratinocytes (Klaassen et al, 1999) or DMEM with 5% FCS for HNSCC cells. As a control retinoid containing medium without cells was included during the incubation period. From the medium two samples of 350 µl were taken and after removal of the residual medium the cells were washed once with phosphate-buffered saline and collected in 350 µl trypsin/EDTA. The samples were stored at –80˚C under nitrogen until retinoid extraction.

### Determination of the turnover rate

Retinoids were analysed by reverse-phase HPLC after extraction with acetonitrile, as described previously (Teerlink et al, 1997; Klaassen et al, 2001a). The amount of retinoid compound at each time point was calculated relative to the amount of the medium control at the start of the experiment. The turnover rate was taken as the difference between 4 and 24 h, corrected for the decrease in the medium controls during this period. Next, this relative amount was converted to absolute amounts in pmol by use of the external standards. The turnover rate was expressed in pmol per mg protein per hour. Protein content of cell extracts was determined on the pellet with the Bio-Rad protein assay (Bio-Rad, Hercules, CA)

### RESULTS

### Growth inhibition of various retinoid compounds in HNSCC cell lines

The relative growth of four HNSCC cell lines treated with ATRA, its isomers (13cRA and 9cRA), and its precursor ROL is illustrated in Figure 1. Two cell lines (UM-SCC-22A and UM-SCC-35) exerted a dose-dependent growth inhibition for all compounds, while the other two cell lines (UM-SCC-14C and UM-SCC-35R) were insensitive; UMSCC-35 was the most sensitive cell line. 4-oxo-metabolites of ATRA and 13cRA were tested in four cell lines to find out whether these catabolites were able to induce growth inhibition and whether a lack of 4-hydroxylation could explain the lack of response in the insensitive cell lines. From Figure 1 it can be seen that the level of growth inhibition by 4-oxo-RA and 4-oxo-13cRA was similar to that of the other retinoid compounds. In addition, OKC cultures from different individuals were exposed to four retinoids as well; a moderate dose-dependent growth inhibition was observed that was not different between the various compounds (Figure 1).

### Metabolism of ATRA, 13cRA, 9cRA and ROL in HNSCC cells and OKC cultures

A summary of the turnover rate of all different compounds and the metabolites formed in the medium and pellets is given in Table 1 and Figure 2. The rate of retinoid turnover was more cell line than compound dependent. In general, UM-SCC-35 showed the highest turnover rate for all compounds, UM-SCC-35R and UM-SCC-14C the lowest and UM-SCC-22A an intermediate turnover rate; turnover rates in OKC were comparable to the turnover rates in UM-SCC-35R and UM-SCC-14C. The turnover rate of ROL was the highest of all compounds in all cell lines.

© 2001 Cancer Research Campaign

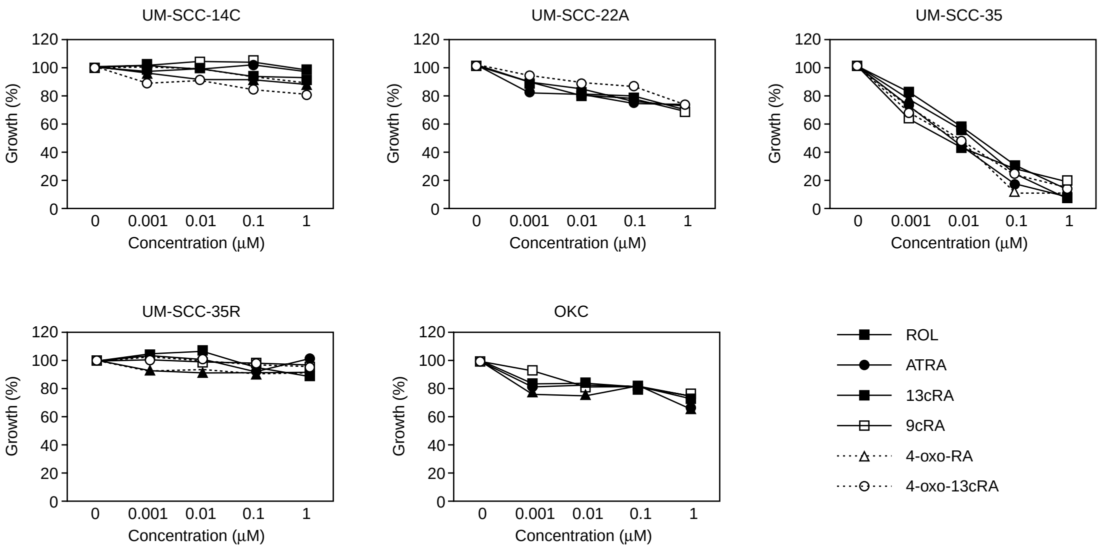

**Figure 1** Relative growth of cells exposed to various concentrations of ATRA, 13cRA, 9cRA, ROL, 4-oxo-13cRA and 4-oxo-RA as compared to untreated cells. OKC cultures (from four different individuals) were exposed to the first four drugs. Growth was assessed with the 'SRB-assay' and experiments were performed at least three times. Average values are shown and coefficients of variation were less than 5% (HNSCC lines) and 13% (OKC cultures), respectively

**Table 1** Retinoid metabolism in four HNSCC cells and OKC exposed to 1 mM of four different retinoids

| HNSCC line | Retinoid | Metabolism* | | Metabolites** | |
|---|---|---|---|---|---|
| | | Mean | SD | Pellet | Medium |
| 14C | ATRA | 13.3 | 9.2 | e | a,b |
| | 13cRA | 78.7 | 27.2 | d2 | a,b |
| | 9cRA | 59.3 | 13.2 | e | a,b |
| | ROL | 223.7 | 84.5 | d1 | d1 |
| 22A | ATRA | 104.8 | 67.9 | e | a,b |
| | 13cRA | 154.1 | 76.3 | d2 | a,b |
| | 9cRA | 139.3 | 45.9 | e | a,b |
| | ROL | 349.9 | 12.7 | d1 | d1 |
| 35 | ATRA | 1,016.0 | 413.2 | e | a,b |
| | 13cRA | 949.5 | 53.0 | e | a,b |
| | 9cRA | 962.3 | 88.2 | b | a,b,c |
| | ROL | 1,336.8 | 76.7 | c1 | a,b,c1,d1 |
| 35R | ATRA | 52.7 | 25.4 | b | a,b |
| | 13cRA | 6.9 | 9.8 | b,d2 | a,b |
| | 9cRA | 23.4 | 5.5 | b | a,b |
| | ROL | 111.7 | 2.9 | c1,d1 | c1,d1 |
| OKC | ATRA | 14.8 | 19.0 | e | e |
| | 13cRA | 38.8 | 20.5 | d2 | e |
| | 9cRA | 12.0 | 12.5 | e | e |
| | ROL | 98.7 | 78.5 | d1 | d1 |

The profiles of 72 h exposure are shown. Note that the names of the cell lines have been abbreviated; the prefix 'UM-SCC-' has been omitted. Experiments were performed in triplicate, OKC cultures were from four different individuals. *Metabolism determined from HPLC analysis expressed as relative decrease in medium and cells together of the corresponding retinoid between 4 and 24 h exposure as compared to medium controls at t = 0 h per mg protein per hour. **Metabolites in medium and in cell pellets determined from HPLC analysis. Metabolites: a, between 5 and 10 min (retention time); b, between 10 and 15 min; c, between 15 min and 13cRA; d, between ATRA and ROL; e, no metabolites. A more detailed explanation of the metabolites mentioned is given in Figure 2.

In general, the metabolites formed were different between HNSCC cell lines and OKC (Table 1). Polar metabolites in 'group *b*' and metabolites in 'group *a*' (Figure 2) were only found in HNSCC cell lines, but not in OKC. The widest variety of metabolites was found in the medium of cell line UM-SCC-35, probably due to its high turnover rate. 5, 6-epoxy-RA was, for instance, only found in medium of UM-SCC-35. After 13cRA and 9cRA exposure, a relative high formation of polar metabolites in UM-SCC-35 and UM-SCC-35R was observed, similar as was reported for ATRA (Klaassen et al, 2001a). Metabolism of ROL resulted in the formation of an unknown product, with a retention time a little longer than that of ATRA (Figure 2, '*d*'), which was found in almost every medium and cell fraction of cancer cell lines as well as OKC, whereas a group of unknown peaks named '*c*1' was only found in

 *© 2001 Cancer Research Campaign*

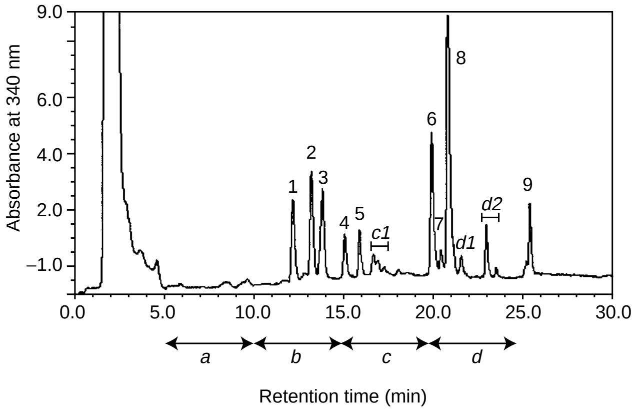

**Figure 2** Compilation of all metabolites formed in cells and medium after addition of ATRA, 9cRA, 13cRA or ROL is shown. This chromatogram is necessary for the interpretation of Table 1 and shows 4-oxo-RA (peak 1), 4-oxo-cRA (2), 4-OH-RA (3), 18-OH-RA (4), 5,6-epoxy-RA (5), 13cRA (6), 9cRA (7), ATRA (8) and ROL (9). Furthermore, the chromatogram is divided in groups of peaks as follows: *a*, polar metabolites corresponding to peaks with retention times between 5 and 10 min; *b*, polar metabolites corresponding to peaks with retention times between 10 and 15 min; *c*, polar metabolites corresponding to peaks with retention times between 15 min and that of 13cRA; *d*, peaks with retention times between that of ATRA and ROL; *c1*, peaks found after exposure to ROL in medium and cells of UM-SCC-35 and UM-SCC-35R; *d1*, peak found after exposure to ROL in medium and cells of HNSCC cell lines and OKC; *d2*, peaks found in cells of HNSCC cell lines and OKC after exposure to 13cRA. Peak 5 (5,6-epoxy-RA) was found only in medium of UM-SCC-35 after exposure to 9cRA

UM-SCC-35 and UM-SCC-35R (Table 1). Another group of unknown peaks named '*d*2', corresponding with retention times between that of ATRA and ROL, was found after exposure to 13cRA in all cellular fractions, except in that of UM-SCC-35. The unidentified peaks described above were not found in medium without cells or in untreated cells.

As an example, profiles of metabolites formed in the medium of UM-SCC-35 after exposure to four retinoids are shown in Figure 3. Each compound corresponds with an unique pattern of metabolites. In the other HNSCC cell lines generally similar peak profiles as in UM-SCC-35 were found. Following ROL and 9cRA exposure some more peaks were detected in UM-SCC-35 and –35R (Table 1). Co-elution of peaks with authentic retinoids of absorption at the two wavelengths of detection with the corresponding ratios of the reference compounds enabled the identification of 4-oxo-RA (1), 4-oxo-13cRA (2), 4-OH-RA (3), 18-OH-RA (4), and an unknown peak (A) after exposure to ATRA (Figure 3). The major metabolite of ATRA in UM-SCC-35 was 4-OH-RA. 13cRA was metabolized to products identified as 4-oxo-13cRA (2) and three unknown peaks (A, B and C), of which peak A and B were the highest (Figure 3). Metabolism of 9cRA resulted in peaks corresponding with the same metabolites as found with 13cRA metabolism, but with higher amounts of peak A and a substantially lower amount of peak B and an additional peak co-eluting with 5, 6-epoxy-RA (peak 5). The level of the polar metabolites (Figure 2, 'group *b*') formed after 13cRA or 9cRA exposure was much higher than that of the group of polar metabolites formed from ATRA.

After ATRA exposure, as compared to other HNSCC cell lines a relatively high isomerization (up to 10%) was found to 13cRA in UM-SCC-22A, UM-SCC-14C and OKC and a relatively high level of polar metabolites in UM-SCC-35R. In UM-SCC-22A, UM-SCC-14C and OKC a relatively high isomerization to ATRA after 13cRA exposure (up to 10%) and a relatively high isomerization to

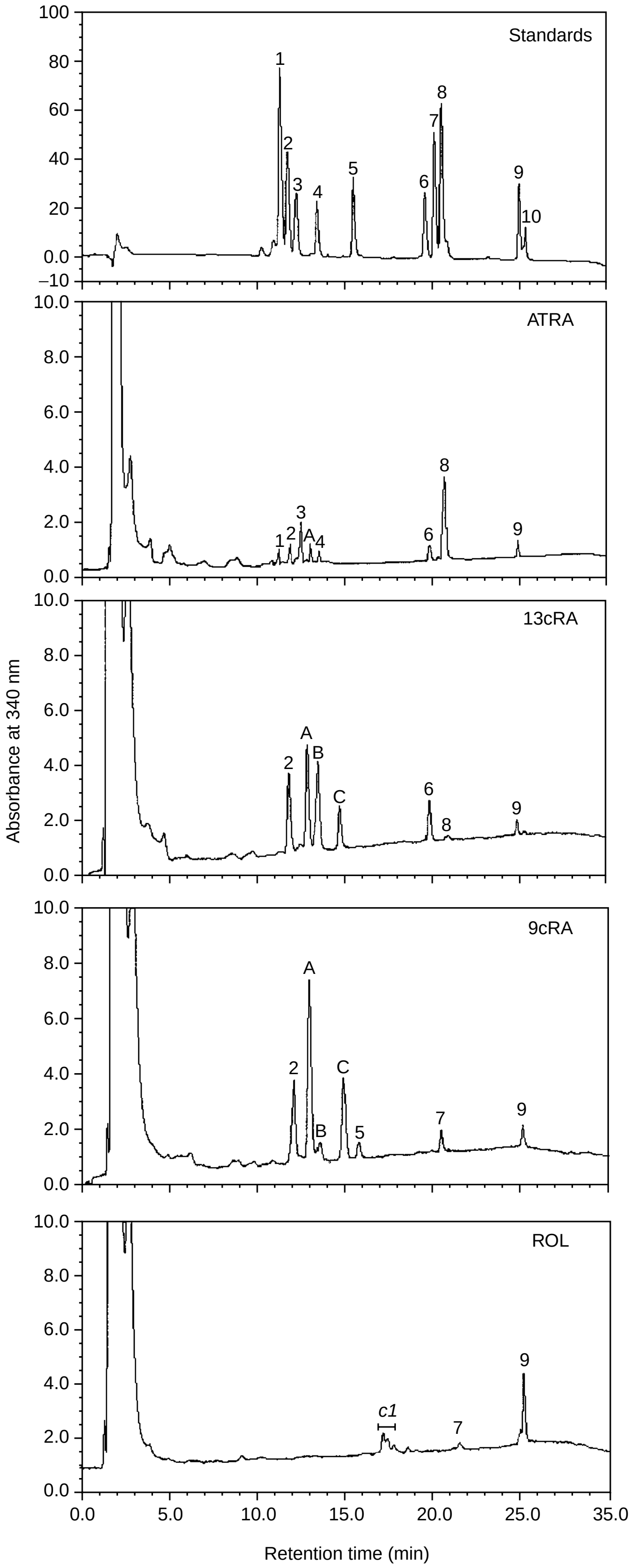

**Figure 3** HPLC profiles of retinoid metabolites in the medium of UM-SCC-35 are shown after exposure to $10^{-6}$ $mol^{-1}$ M of various retinoid compounds for 24 h. The upper panel one is a HPLC profile of retinoid standards dissolved in water. For identification of the peaks see the legend of Figure 2 and in addition peak 10 corresponds to retinal. Peaks A, B and C indicate unidentified compounds

© 2001 Cancer Research Campaign

ATRA and 13cRA after 9cRA exposure (up to 5%) was found. After exposure to ROL, a relatively high formation of ATRA in UM-SCC-14C was seen and no or very small amounts of other retinoids in UM-SCC-35 and UM-SCC-35R.

### DISCUSSION

Treatment with 13cRA has shown several major drawbacks: it exhibited considerable toxicity at therapeutic levels, was active in only a proportion of the individuals with premalignant lesions and when the treatment is stopped the lesions recur (Hong et al, 1986; Hong et al, 1990). Other retinoic acid derivatives are being developed and tested, hoping that these derivatives may lack the negative effects of 13cRA and be able to provide similar or higher efficacy, including activity against advanced cancer (Armstrong and Meyskens, 2000). Recently, a randomized Phase I chemoprevention trial with ATRA in patients with curatively treated HNSCC ended and a Phase II trial is on its way (Park et al, 2000).

The present study showed that all retinoids tested were equally active in HNSCC cell lines. Thus, although retinoids have different binding affinities for the nuclear receptors (Allenby et al, 1993; Sun et al, 1997), this apparently has no influence on the level of growth inhibition in vitro. This finding suggests that all these compounds induce growth inhibition through a common mechanism. Evidence has been provided that retinoids regulate cellular growth and differentiation through binding to retinoic acid receptors (RARs) and retinoid X receptors (RXRs) (Mangelsdorf et al, 1994; Heyman et al, 1992). In particular, in HNSCC the binding to RAR-γ has been suggested to be important for retinoid induced growth inhibition. (Le et al, 2000; Klaassen et al, 2001b). So the most likely explanation is that all retinoids are able, in a direct or indirect way, to reach the downstream target, the retinoid receptor. It remains to be explained why 13cRA is active in some HNSCC lines whereas the binding affinity of 13cRA to both retinoic acid receptor classes is negligible. It is very plausible that 13cRA is converted to retinoids that have high enough levels to modulate growth through binding to the retinoid receptors. 13cRA is converted to its isomers ATRA and 9cRA and its breakdown product 4-oxo-13cRA; all these compounds have receptor binding property and a growth inhibiting activity (Figure 1).

The retinoids used in this study were not able to improve the sensitivity profile of the standard compound 13cRA. In the insensitive cell lines ATRA, 9cRA and ROL did not enhance the level of growth inhibition. It is possible that in these cell lines the receptor system is seriously disrupted and that it will be very hard to find a retinoid that will be active.

We tested turnover rates of 13cRA, ATRA, 9cRA and ROL to investigate whether these would explain differences in growth inhibition between HNSCC cell lines. In the individual cell lines, turnover rates of the three stereoisomers were in a narrow range, and that of ROL somewhat higher. The most likely explanation for the relatively higher turnover rate of ROL as compared to the other retinoids might be that ROL can be converted to at least four different compounds. Besides oxidation to ATRA via retinal, ROL can also be esterified to retinyl-esters (Kurlandsky et al, 1996), 4-hydroxylated to 4-oxo-ROL (Leo and Lieber, 1985), or metabolized to anhydroretinol (Bhat et al, 1979). Esterification has been studied in more detail by Guo and Gudas (1998). These authors showed that HNSCC cells have a reduced capacity to esterify ROL as compared to normal oral keratinocytes. This would explain the relatively low turnover rate of ROL as compared to tumour cell cultures, but whether this leads to differences in metabolites, remains to be established.

We also performed metabolic studies on normal OKC. These cells were slow with respect to the rate of retinoid turnover and were not able to synthesize polar metabolites. This confirms the results of a previous study with ATRA (Klaassen et al, 2001a).

The comparison of the retinoids with respect to metabolism showed two phenomena that need further discussion. There was only a small difference between the compounds in turnover rate and each compound had its unique pattern of metabolites. With respect to the rate of turnover it is known that the CYP-related enzyme system is involved in the conversion (4-hydroxylation) to polar retinoids, like 4-OH-RA, 18-OH-RA and 4-oxo-RA. For instance, CYP26 was found to be responsible for the 4-hydroxylation of ATRA (Ray et al, 1997; White et al, 1997). CYP26 is highly substrate (ATRA) specific and can not metabolize 13cRA or 9cRA (Sonneveld et al, 1998). In the present study it was found that 13cRA and 9cRA were catabolized with similar turnover rates as ATRA, which suggests that one or more enzymes analogous to CYP26 are responsible for the catabolism of 13cRA and 9cRA. To a small degree, isomerization to ATRA can be involved, however the finding that each retinoid has its unique pattern of metabolites limits this possibility. Furthermore, other studies have shown evidence that 4-hydroxylated forms of 9cRA and 13cRA are directly formed from their parental compounds (Eckhoff and Nau, 1990; Dzerk et al, 1998). This strongly suggests that specific CYPs exist that are responsible for the conversion of 9cRA and 13cRA to their corresponding 4-oxo-compounds. The CYP isoform that metabolizes *cis*- and *trans*-RA-isomers can be different as shown with isomers of retinal. Raner et al (1996) demonstrated that in vitro 4-hydroxylation of 13-*cis*- and all-*trans*-retinal was predominantly catalysed by the CYP1A1 isoform, whereas CYP2B4 and CYP2C3 are most active in the metabolism of 9-*cis*-retinal.

In conclusion, despite variation in their binding to retinoid receptors, all retinoids did not differ with respect to their capacity to induce growth inhibition. The rate at which the cells were able to catabolize the retinoid was similar for all compounds. Cellular metabolism in HNSCC cells leads to a metabolite profile that is unique for each retinoid.

### ACKNOWLEDGEMENTS

This work was supported by the Dutch Cancer Society, grant 95–926.

### REFERENCES

Allenby G, Bocquel MT, Saunders M, Kazmer S, Speck J, Rosenberger M, Lovey A, Kastner P, Grippo JF, Chambon P and Levin AE (1993) Retinoic acid receptors and retinoid X receptors: interactions with endogenous retinoic acids. *Proc Natl Acad Sci USA* **90**: 30–34

Armstrong WB and Meyskens FL Jr (2000) Chemoprevention of head and neck cancer. *Otolaryngol Head Neck Surg* **122**: 728–735

Åström A, Pettersson U, Krust A, Chambon P and Voorhees JJ (1990) Retinoic acid and synthetic analogs differentially activate retinoic acid receptor dependent transcription. *Biochem Biophys Res Commun* **173**: 339–345

Bhat PV, De Luca LM, Adamo S, Akalovsky I, Silverman-Jones CS and Peck GL (1979) Retinoid metabolism in spontaneously transformed mouse fibroblasts (Balb/c 3T12–3 cells): enzymatic conversion of retinol to anhydroretinol. *J Lipid Res* **20**: 357–362

Bollag W and Holdener EE (1992) Retinoids in cancer prevention and therapy. *Ann Oncol* **3**: 513–526

Braakhuis BJM, Jansen G, Noordhuis P, Kegel A and Peters GJ (1993) Importance of pharmacodynamics in the in vitro antiproliferative activity of the antifolates

 *© 2001 Cancer Research Campaign*

methotrexate and 10-ethyl-10-deazaaminopterin against human head and neck squamous cell carcinoma. *Biochem Pharmacol* **46**: 2155–2161

Braakhuis BJM, Klaassen I, van der Leede BM, Cloos J, Brakenhoff RH, Copper MP, Teerlink T, Hendriks HF, van der Saag PT and Snow GB (1997) Retinoid metabolism and all-trans retinoic acid-induced growth inhibition in head and neck squamous cell carcinoma cell lines. *Br J Cancer* **76**: 189–197

Carey TE, Wolf GT, Baker SR and Krause CJ (1990) Cell surface antigen expression and prognosis. In: *Head and neck cancer*, 2nd edn, Fee WE, Goephert H, Johns ME, Strong EW, and Ward PH (eds) pp 77–82. BC Decker: Toronto – Philadelphia

Dzerk AM, Carlson A, Loewen GR, Shirley MA and Lee JW (1998) A HPLC method for the determination of 9-cis retinoic acid (ALRT1057) and its 4-oxo metabolite in human plasma. *J Pharm Biomed Anal* **16**: 1013–1019

Eckhoff C and Nau H (1990) Identification and quantitation of all-trans-and 13-cis-retinoic acid and 13-cis-4-oxoretinoic acid in human plasma. *J Lipid Res* **31**: 1445–1454

Gudas LJ, Sporn MB and Roberts AB (1994) Cellular biology and biochemistry of the retinoids. In: *The Retinoids*, 2nd edn, Sporn MB, Roberts AB and Goodman DS (eds) pp 443–520. Raven Press: New York

Guo X and Gudas LJ (1998) Metabolism of all-trans-retinol in normal human cell strains and squamous cell carcinoma (SCC) lines from the oral cavity and skin: reduced esterification of retinol in SCC lines. *Cancer Res* **58**: 166–176

Heyman RA, Mangelsdorf DJ, Dyck JA, Stein RB, Eichele G, Evans RM and Thaller C (1992) 9-cis retinoic acid is a high affinity ligand for the retinoid X receptor. *Cell* **68**: 397–406

Hong WK, Endicott J, Itri LM, Doos W, Batsakis JG, Bell R, Fofonoff S, Byers R, Atkinson EN, Vaughan C et al (1986) 13-cis-retinoic acid in the treatment of oral leukoplakia. *N Engl J Med* **315**: 1501–1505

Hong WK, Lippman SM, Itri LM, Karp DD, Lee JS, Byers RM, Schantz SP, Kramer AM, Lotan R, Peters LJ, Dimery IW, Brown BW and Goepfert H (1990) Prevention of second primary tumors with isotretinoin in squamous-cell carcinoma of the head and neck. *N Engl J Med* **323**: 795–780

Hong WK and Itri LM (1994) Retinoids and human cancer. In: *The Retinoids*, 2nd edn, Sporn MB, Roberts AB, Goodman DS (eds) pp 597–630. Raven Press: New York

Klaassen I, Brakenhoff RH, Smeets SJ, Snow GB and Braakhuis BJ (1999) Considerations for in vitro retinoid experiments: importance of protein interaction. *Biochem Biophys Acta* **1427**: 265–275

Klaassen I, Brakenhoff RH, Smeets SJ, Snow GB and Braakhuis BJ (2001a) Enhanced turnover of all-trans-Retinoic acid and increased formation of polar metabolites in head and neck squamous cell carcinoma cells compared to normal oral keratinocytes. *Clin Cancer Res* **7**: 1017–1025

Klaassen I, Brakenhoff RH, Smeets SJ, Snow GB and Braakhuis BJ (2001b). The expression of retinoic acid receptor γ correlates with retinoic acid sensitivity and metabolism in head and neck squamous cell carcinoma cell lines. *Int J Cancer* **92**: 661–665

Koch HF (1978) Biochemical treatment of precancerous oral lesions: the effectiveness of various analogues of retinoic acid. *J Maxillofac Surg* **6**: 59–63

Kurlandsky SB, Duell EA, Kang S, Voorhees JJ and Fisher GJ (1996) Auto-regulation of retinoic acid biosynthesis through regulation of retinol esterification in human keratinocytes. *J Biol Chem* **271**: 15346–15352

Le Q, Dawson, MI, Soprano DR and Soprano KJ (2000) Modulation of retinoic acid receptor function alters the growth inhibitory response of oral SCC cells to retinoids. *Oncogene* **19**: 1457–1465

Leo MA and Lieber CS (1985) New pathway for retinol metabolism in liver microsomes. *J Biol Chem* **260**: 5228–5231

Lippman SM, Kessler JF, AI Sarraf M, Alberts DS, Itri LM, Mattox D, Von Hoff DD, Loescher L and Meyskens FL (1988) Treatment of advanced squamous cell carcinoma of the head and neck with isotretinoin: a phase II randomized trial. *Invest New Drugs* **6**: 51–56

Lotan R (1980) Effects of vitamin A and its analogs (retinoids) on normal and neoplastic cells. *Biochim Biophys Acta* **605**: 33–91

Mangelsdorf DJ (1994) Vitamin A receptors. *Nutr Rev* **52**: S32–S44

Muindi JR, Young CW and Warrell RP Jr (1994) Clinical pharmacology of all-trans retinoic acid. *Leukemia* **8** (Suppl 3): S16–S21

Oridate N, Esumi N, Lotan D, Hong WK, Rochette-Egly C, Chambon P and Lotan R (1996) Implication of retinoic acid receptor gamma in squamous differentiation and response to retinoic acid in head and neck SqCC/Y1 squamous carcinoma cells. *Oncogene* **12**: 2019–2028

Park SH, Gray WC, Hernandez I, Jacobs M, Ord RA, Sutharalingam M, Smith RG, Van Echo DA, Wu S and Conley BA (2000) Phase I trial of all-trans retinoic acid in patients with treated head and neck squamous carcinoma. *Clin Cancer Res* **6**: 847–854

Raner GM, Vaz AD and Coon MJ (1996) Metabolism of all-trans, 9-cis, and 13-cis isomers of retinal by purified isozymes of microsomal cytochrome P450 and mechanism-based inhibition of retinoid oxidation by citral. *Mol Pharmacol* **49**: 515–522

Ray WJ, Bain G, Yao M and Gottlieb DI (1997) CYP26, a novel mammalian cytochrome P450, is induced by retinoic acid and defines a new family. *J Biol Chem* **272**: 18702–18708

Reid CB, Cloos J, Snow GB and Braakhuis BJ (1997) A simple and reliable technique for culturing of human oral keratinocytes and fibroblasts. *Acta Otolaryngol* **117**: 628–633

Roberts AB, Lamb LC and Sporn MB (1980) Metabolism of all-trans-retinoic acid in hamster liver microsomes: oxidation of 4-hydroxy- to 4-keto-retinoic acid. *Arch Biochem Biophys* **199**: 374–383

Rockley NL, Halley BA and Nelson EC (1980) Identification of a decarboxylation product of retinoic acid. *Biochem Biophys Acta* **627**: 270–275

Sass JO, Forster A, Bock KW and Nau H (1994) Glucuronidation and isomerization of all-trans- and 13-cis-retinoic acid by liver microsomes of phenobarbital-or 3-methylcholanthrene-treated rats. *Biochem Pharmacol* **47**: 485–492

Sonneveld E, van den Brink CE, van der Leede BM, Schulkes RK, Petkovich M, van der Burg B and van der Saag PT (1998) Human retinoic acid (RA) 4-hydroxylase (CYP26) is highly specific for all-trans-RA and can be induced through RA receptors in human breast and colon carcinoma cells. *Cell Growth Differ* **9**: 629–637

Sun SY, Yue P, Dawson MI, Shroot B, Michel S, Lamph WW, Heyman RA, Teng M, Chandraratna RA, Shudo K, Hong WK and Lotan R (1997) Differential effects of synthetic nuclear retinoid receptor-selective retinoids on the growth of human non-small cell lung carcinoma cells. *Cancer Res* **57**: 4931–4939

Teerlink T, Copper MP, Klaassen I and Braakhuis BJ (1997) Simultaneous analysis of retinol, all-trans-and 13-cis-retinoic acid and 13-cis-4-oxoretinoic acid in plasma by liquid chromatography using on-column concentration after single-phase fluid extraction. *J Chromatogr B Biomed Sci Appl* **694**: 83–92

White JA, Beckett-Jones B, Guo YD, Dilworth FJ, Bonasoro J, Jones G and Petkovich M (1997) cDNA cloning of human retinoic acid-metabolizing enzyme (hP450RAI) identifies a novel family of cytochromes P450. *J Biol Chem* **272**: 18538–18541

© 2001 Cancer Research Campaign 
